# Continual Sequence Modeling With Predictive Coding

**DOI:** 10.3389/fnbot.2022.845955

**Published:** 2022-05-23

**Authors:** Louis Annabi, Alexandre Pitti, Mathias Quoy

**Affiliations:** ^1^UMR8051 Equipes Traitement de l'Information et Systemes (ETIS), CY University, ENSEA, CNRS, Cergy-Pontoise, France; ^2^IPAL CNRS Singapore, Singapore, Singapore

**Keywords:** predictive coding, continual learning, Reservoir Computing (RC), recurrent neural networks (RNN), conceptors

## Abstract

Recurrent neural networks (RNNs) have been proved very successful at modeling sequential data such as language or motions. However, these successes rely on the use of the backpropagation through time (BPTT) algorithm, batch training, and the hypothesis that all the training data are available at the same time. In contrast, the field of developmental robotics aims at uncovering lifelong learning mechanisms that could allow embodied machines to learn and stabilize knowledge in continuously evolving environments. In this article, we investigate different RNN designs and learning methods, that we evaluate in a continual learning setting. The generative modeling task consists in learning to generate 20 continuous trajectories that are presented sequentially to the learning algorithms. Each method is evaluated according to the average prediction error over the 20 trajectories obtained after complete training. This study focuses on learning algorithms with low memory requirements, that do not need to store past information to update their parameters. Our experiments identify two approaches especially fit for this task: conceptors and predictive coding. We suggest combining these two mechanisms into a new proposed model that we label PC-Conceptors that outperforms the other methods presented in this study.

## 1. Introduction

Continual learning is a branch of machine learning aiming at equipping learning agents with the ability to learn incrementally without forgetting previously acquired knowledge. The continual learning setting typically involves several separate tasks where we assume data to be independent and identically distributed. The learning algorithm is confronted with each source of data (i.e., each task) sequentially. After a set amount of time on a task, training switches to a new task. This process is repeated until the learning algorithm has been confronted with all tasks.

Learning methods based on iterative updates of model parameters, such as the backpropagation algorithm, can be performed sequentially as new data becomes available. However, these methods might suffer from the problem known as catastrophic forgetting (McCloskey and Cohen, 1989) if the distribution of the data they process evolves over time. When adapting to the new task, they automatically overwrite the model parameters that were optimized according to the previous tasks. This is an important issue since it prevents artificial neural networks from being trained incrementally.

In this study, we focus on the problem of learning a repertoire of trajectories. As such, the training examples in each task are sequences that the learning algorithm has to generate from a discrete input (i.e., the index of the sequence). We study different Recurrent Neural Network (RNN) designs and learning algorithms for this continual learning task. We limit our comparison to models with low memory requirements and, thus, impose that at each time step *t*, the neural network computations and learning can only access the currently available quantities. In our case, these quantities are the currently hidden variables of the models, and the target output $x_{t}^{*}$ provided by the data set. Consequently, learning methods based on BPTT do not qualify for this criterion, as they need to store in memory the past activations of the RNN hidden states to compute gradients. The advantage of models fitting this criterion is that they could in principle be implemented on dedicated hardware reproducing the neural network architecture, with no need for an external memory storing past inputs and activations.

To avoid confusion about the use of the word “online,” we rather talk about *continual* learning to refer to the task temporality, and talk about *online* learning to refer to the sequence (the training example) temporality. The models studied in this section are thus trained both in a continual learning setting, since the different target trajectories are provided sequentially to the agent, and using online learning mechanisms since the algorithms for learning do not rely on a memory of past activations. The article is structured as follows: in Section 2, we review methods that have been proposed to mitigate the problem of catastrophic forgetting in artificial neural networks, as well as learning algorithms that can be performed online. In Section 3, we describe the experimental setting and the different algorithms, and present the obtained results in Section 4. Finally, in Section 5, we discuss our results in order to identify the online learning mechanisms for RNNs most suited for the continual learning of a repertoire of sequences.

## 2. Related Work

There exists a large spectrum of methods to mitigate catastrophic forgetting in continual learning settings. Regularization methods typically aim at limiting forgetting by constraining learning with, e.g., sparsity constraints, early stopping, or identified synaptic weights that should not be overwritten. For instance, in Elastic Weight Consolidation (EWC) (Kirkpatrick et al., 2017), the update rule contains a regularization term that pulls the synaptic weights toward the optimal weights found for previous tasks, with a strength depending on the estimated importance of each synaptic weight.

Another approach is to rely on architecture modifications when new tasks are presented, for instance by freezing some of the previously learned weights (Mallya et al., 2018), or by adding new neurons and synaptic connections to the model (Li and Hoiem, 2017). Finally, rehearsal (Rebuffi et al., 2017) and generative replay (Shin et al., 2017) methods rely on saving examples or modeling past tasks for future use. By inserting training examples from the previous tasks, either saved or replayed, into the current task, these methods allow to retrain on those data points and thus limiting catastrophic forgetting.

In this study, we compare learning algorithms with low memory requirements in a continual learning setting. As such, we disregard approaches such as rehearsal and generative replay and only consider some simple regularization or architectural techniques to improve the performance of sequence memory models in a continual learning setting.

Many alternatives to BPTT have been investigated in the past decades, often with the goal of avoiding the problems known as exploding and vanishing gradients that can arise when using this learning algorithm (Pascanu et al., 2013). Here, we study two alternative approaches, namely, learning with evolution strategies, and Reservoir Computing (RC) (Verstraeten et al., 2007; Lukosevicius and Jaeger, 2009).

Using evolution strategies allows for learning RNN parameters without having to rely on past activations. The success of a certain parameter configuration can be measured online, for instance by comparing the network's output at each time step *t* with the target output. Then, this score is used as the fitness measure to be minimized by evolution. Following this approach, Schmidhuber et al. (2005) and Schmidhuber et al. (2007) co-evolve different groups of neurons in a Long Short-Term Memory (LSTM) network. A similar approach is used by Pitti et al. (2017), where the fitness measure is used to directly optimize the inputs of an RNN.

Completely avoiding the problem of learning recurrent weights, a family of approaches has emerged in parallel with the field of computational neurosciences in the form of Liquid State Machines (Maass et al., 2002), and from the field of machine learning in the form of Echo State Networks (ESN) (Jaeger, 2001). These models, later brought together under the label of Reservoir Computing (Verstraeten et al., 2007; Lukosevicius and Jaeger, 2009), discards the difficulties of learning recurrent weights by instead developing techniques to find relevant initializations of these parameters.

Typically, the recurrent connections are set in order for the RNN to exhibit rich non-linear (and sometimes self-sustained) dynamics, that are decoded by a learned readout layer. If the dynamics of the RNN activation are complex enough (e.g., they do not converge too rapidly toward a point attractor or limit cycle attractor), various output sequences can be decoded from those. Training RC models then come down to learning the weights of the readout layer, which is an easier optimization problem that can be tackled with several algorithms. This output layer can, e.g., be trained using stochastic gradient descent, without the need for BP. The FORCE algorithm (Sussillo and Abbott, 2009) improves this learning by running an iterative estimate of the correlation matrix of the hidden state activations.

Another interesting learning mechanism is presented in Jaeger (2014a,b) under the name of Conceptors. This method exploits the fact that the hidden state dynamics triggered by an input pattern is typically bounded to a certain subspace of lower dimension. By identifying the subspace for each possible input pattern, it is possible to decorrelate the training of each target trajectory by focusing learning on the readout connections that come from the corresponding hidden state subspace (called Conceptor). This method allows training a sequence memory where the learning of a new pattern has limited interference with already learned ones.

Finally, the Predictive Coding (PC) theory (Rao and Ballard, 1999; Clark, 2013) also provides learning rules that do not rely on past activations. According to PC, prediction error neurons are intertwined with the neural generative model and encode at each layer the discrepancy between the top-down prediction and the current representation. It has been shown that this construction allows propagating the output prediction error information back into the generative model and even approximates the backpropagation algorithm (Whittington and Bogacz, 2017; Millidge et al., 2020).

Taking inspiration from the PC theory, we propose several models that integrate prediction error neurons into a simple RNN design. These prediction error neurons transport the error information from the output layer to the hidden layer, which provides a local target that can be used to learn the recurrent and input weights. We label the resulting models PC-RNN (for Predictive Coding Recurrent Neural Network). In Appendix A, we show how these models can be derived from the application of gradient descent on a quantity called variational free-energy expressed according to different generative models. The resulting models slightly deviate from other approaches such as the Parallel Temporal Coding Network (P-TNCN) described in Ororbia et al. (2020), and the original PC model presented in Rao and Ballard (1997), which suggests learning feedback weights responsibly for the bottom-up computations instead of copying the forward weights, as performed in the proposed models.

There have been other evaluations of continual learning methods applied to RNNs (Sodhani et al., 2020; Cossu et al., 2021b), some even focusing on ESNs (Cossu et al., 2021a). While these studies compare many continual learning techniques, they do not consider the online learning constraint, and almost exclusively focus on sequence classification tasks. In contrast, this study investigates continual learning methods that can be used online, applied to the incremental learning of a repertoire of trajectories.

## 3. Materials and Methods

In this section, we detail our experimental setting as well as the different models that we use for the comparative study.

### 3.1. Experimental Setting

**Figure 1:**
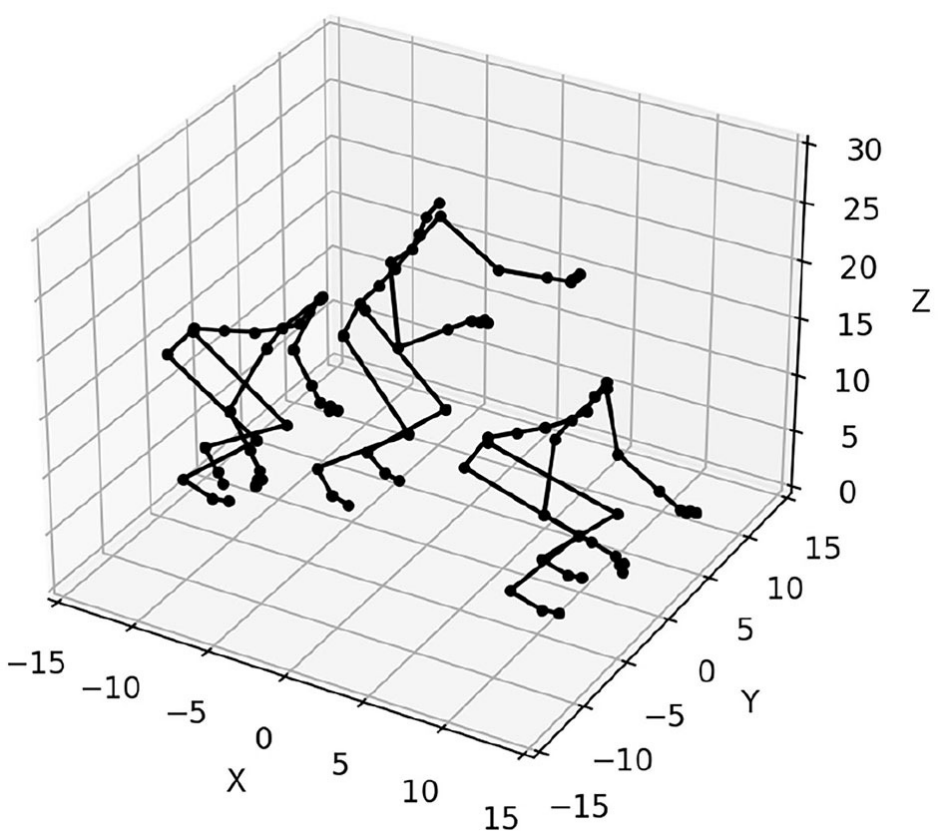
Three body configurations taken from a trajectory capturing a jump motion.

Each RNN model is trained sequentially on *p* sequence generation tasks. The *p* sequences to be learned are sampled from a data set of motion capture trajectories of dimension 62. Each point $x_{t}^{*}$ describes a body configuration, as represented in Figure 1. These trajectories were obtained from the CMU Motion Capture Database. We make a distinction between the validation set used to optimize the hyperparameters of each model, and the test set, used to measure the performance of each model. In our experiments, the validation set is composed of *p* = 15 trajectories of a subject (#86 in the database) practicing various sports. The test set is composed of *p* = 20 trajectories of a subject (#62 in the database) performing construction work (screwing, hammering, etc.).

We also measure the performance of each model on a different test set of *p* = 20 simple 2D trajectories corresponding to handwritten letters taken from the UCI Machine Learning Repository (Dua and Graff, 2019). All trajectories are resampled to last for 60 time steps. These data sets were chosen since they represent potential use cases of the models presented in this work. For instance, the proposed continual learning algorithms could be used to incrementally train a robot manipulator to perform certain motor trajectories.

We assume that the model knows when a transition between two tasks occurs, and provide to the RNN the current task index *k* as a one-hot vector input of dimension *p*. Otherwise, this distributional shift could, e.g., be automatically detected through a significant increase of the prediction error.

The end goal of this experiment is to identify online learning mechanisms for RNNs that extend properly to the continual learning case. The RNN architectures typically comprise three types of weight parameters to be learned: the output weights, the recurrent weights, and the input weights. As such, we split our analysis into three comparisons focusing on the learning methods for each type of parameter.

For each learning mechanism, we perform an optimization of hyperparameters using Bayesian optimization with Gaussian processes and Matern 5/2 Kernel, similarly to the RNN encoding capacity comparative analysis performed in Collins et al. (2016).

This method tries to approximate the function ***P***→*f*(***P***) that associates a scoring function with a certain hyperparameter configuration ***P***. This approximation is estimated based on points (***P***_0_, *f*(***P***_0_)), (***P***_1_, *f*(***P***_1_)), ⋯ sampled sequentially by the optimizer. The function used by the optimizer to guide its sampling process is called acquisition function. Here, we used an expected improvement acquisition function, meaning that at each iteration, the optimizer samples the point *P* which is most likely to improve the current estimated maximum of the function *f*. Compared to exhaustive hyperparameter optimization methods such as random search or grid search, this method is expected to converge faster and to better configurations. To perform this hyperparameter optimization we used the gp_minimize function from the scikit-optimize library in python.

The hyperparameters of the models are optimized in order to minimize the final average prediction error on the *p* target sequences of the validation set. For each model, the hyperparameters to optimize are the learning rates associated with the input, recurrent, and output weights, as well as some other coefficients specific to certain learning algorithms. The score function associates each hyperparameter configuration with a real-valued score computed as the negative logarithm of the average prediction error at the end of training.

With the hyperparameter configurations we obtain, we perform for each model 10 seeds of training in the continual learning setting to measure their performances. The final average prediction error on the *p* sequences can be used to evaluate and compare the different learning mechanisms.

### 3.2. Benchmark Models

The models for this benchmark were chosen in order to identify the relevant mechanisms for training RNNs in a continual learning setting. As already said, we also limit this analysis to learning algorithms that can be performed *online*, i.e., without relying on past activations. For each set of weights, we compare the different models listed in Table 1.

**Table 1:** Summary of the models used in our benchmark.

**Weights**	**Model**
Output weights	ESN (Widrow-Hoff)
	Conceptors
	EWC
	ESN + GR
Recurrent weights	PC-RNN-V
	P-TNCN
	PC-RNN-Hebb
Input weights	PC-RNN-HC-A
	PC-RNN-HC-M
	PC-RNN-HC-A-RS
	PC-RNN-HC-M-RS

#### 3.2.1. Output Weights

**Figure 2:**
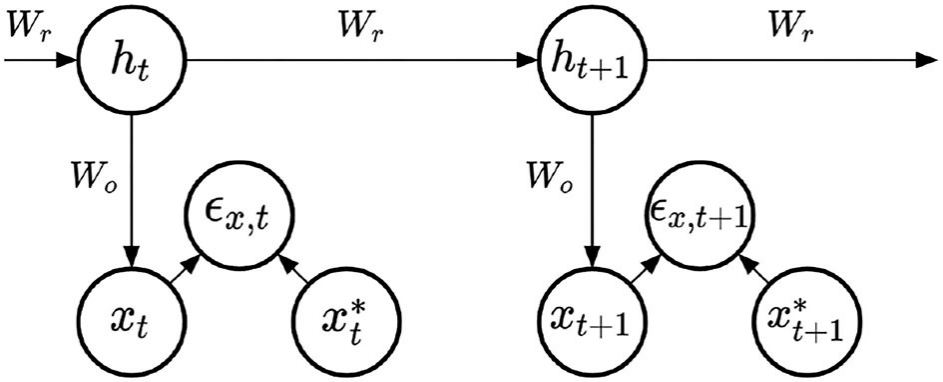
Simple RNN model.

For the learning of the output weights of RNNs, denoted ***W***_*o*_, we compared four learning methods, applied to the simple RNN architecture represented in Figure 2. All methods share the same architecture, and do not provide any learning mechanism for the recurrent weights. At each time step, the hidden state and output prediction are obtained with the following equations:


(1) $$\begin{array}{rcl} h_{t} & = & \left(1-\frac{1}{\tau }\right)h_{t-1}+\frac{1}{\tau }W_{r}\cdot \tanh\left(h_{t-1}\right) \end{array}$$

(2) $$\begin{array}{rcl} x_{t} & = & W_{o}\cdot \tanh\left(h_{t}\right) \end{array}$$

where τ is a time constant controlling the velocity of the hidden state dynamics.

The four methods differ with regard to the learning mechanism applied to the output weights. First, output weights can be learned using standard stochastic gradient descent. In the RNN models we consider, the prediction ***x***_*t*_ is not re-injected into the recurrent computations. As such, the output weights gradients can be computed using only the target signal $x_{t}^{*}$, the prediction ***x***_*t*_, and the hidden state ***h***_*t*_. These computations do not involve the backpropagation of a gradient through time and thus qualify as an online learning method. This first learning rule, also known as the Widrow-Hoff learning rule is expressed as:


(3) $$\begin{array}{r} W_{o}\leftarrow W_{o}+\lambda \epsilon_{x,t}\cdot \tanh{\left(h_{t}\right)}^{⊺} \end{array}$$

where λ is the learning rate of the output weights, and ***ϵ***_*x, t*_ is the prediction error on the output layer, i.e., the difference $x_{t}^{*}-x_{t}$.

The second learning mechanism that we study is stochastic gradient descent aided by Conceptors (Jaeger, 2014a,b). Mathematically, this method can be implemented using only online computations. The Conceptor ***C*** associated with some input can be defined as the matrix corresponding to a soft projection on the subspace where the hidden state dynamics lie when stimulated with this input. The softness of this projection is controlled by a positive parameter α called the aperture. This matrix ***C*** can be computed using the hidden state correlation matrix ***R*** estimated online based on the hidden state dynamics:


(4) $$\begin{array}{r} C=R\cdot {\left(R+\alpha^{-2}I\right)}^{-1} \end{array}$$

(5) $$R_{t+1}=(1-\frac{1}{t+1})R_{t}+\frac{1}{t+1}(h_{t}\;\cdot \;h_{t}^{⊺})$$

In a continual learning setting, for each new task, we can compute the Conceptor corresponding to the complement of the subspace where the previously seen hidden states lie, as 𝕀−***C***. This Conceptor is used to project the new hidden states into a subspace orthogonal to the subspace in which lie the previously seen hidden states. Learning is then performed only on the synaptic weights involving the components of this subspace:


(6) $$W_{o}\leftarrow W_{o}+\text{λ}\epsilon_{x,t}\;\cdot \;{\left(\left(I-C\right)\;\cdot \;\tanh(h_{t})\right)}^{⊺}$$

As shown in Equation 4, low values of α induce a Conceptor matrix close to 0, leading to a projection matrix (𝕀−*C*) close to the identity. On the opposite, high values of α induce a conceptor matrix close to the identity matrix, leading to a hard projection hindering learning.

The third learning mechanism that we study is the Elastic Weight Consolidation (EWC) (Kirkpatrick et al., 2017) algorithm applied to the output weights of the RNN. On each task *k*, we can compute the Fischer information matrix ***F***_*k*_, where each coefficient *F*_*k, i*_ measures the importance of the synaptic weight *W*_*o, i*_:


(7) $$F_{k,i}=\sum_{t=1}^{T}(\nabla_{W_{o,i}}\|x_{t}-x_{k,t}^{*}{\|}_{2}^{2}{)}^{2}$$

where $x_{k,t}^{*}$ denotes the target at time *t* for the task *k*. Then, on a new task *k*′, EWC minimizes the following loss function for each synaptic weight *W*_*o, i*_:


(8) $$\begin{array}{r} L\left(W_{o,i}\right)=L_{k^{\prime }}\left(W_{o,i}\right)+\underset{k<k^{\prime }}{\sum }\frac{\beta }{2}F_{k,i}{\left(W_{o,i}-W_{k,i}^{*}\right)}^{2} \end{array}$$

where $L_{k^{\prime }}$ denotes the loss for task *k*′ without EWC regularization, β is a hyperparameter controlling the importance of the new task with regard to previous tasks, and $W_{k,i}^{*}$ denotes the *i*-th component of the optimal synaptic weights $W_{k}^{*}$ learned on task *k*. We optimize this loss function using gradient descent on the synaptic weights ***W***_*o*_, and obtain the following learning rule:


(9) $$\begin{array}{l} W_{o}\leftarrow W_{o}+\text{λ}\epsilon_{x,t}\cdot \tanh{\left(h_{t}\right)}^{⊺}\\ \;-\text{λ}\beta \left[\left(\sum_{k<k^{\prime }}F_{k}\right)⊙W_{o}-\sum_{k<k^{\prime }}F_{k}⊙W_{k}^{*}\right] \end{array}$$

We can observe that the second line pulls ***W*_*o*_** toward the optimal output weights found for previous tasks, weighted by coefficients measuring the importance of each synaptic weight. In terms of memory requirements, we need to store the sum of the Fischer matrices, as well as the sum of previous optimal synaptic weights weighted by the fisher matrices.

Finally, we also experiment with Generative Replay (GR) as a continual learning technique mitigating catastrophic forgetting. Since each individual task consists precisely in learning to generate the task data (the trajectory), the learned generative model can directly be used to provide samples of the previous tasks. We apply this technique to the simple ESN model described beforehand. At each new task *k*′, we create a copy of the model trained on the tasks *k*<*k*′. This copy is used to generate samples {***x***_1_, ***x***_*T*_} that should be close to the previous tasks' trajectories. During training on the task *k*′, the ESN is also trained in parallel to predict these replayed trajectories, which mitigates catastrophic forgetting.

#### 3.2.2. Recurrent Weights

**Figure 3:**
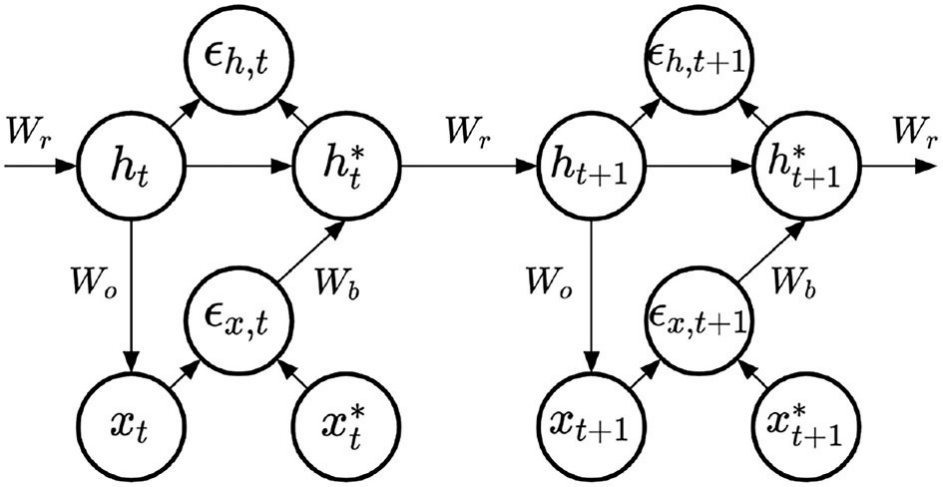
PC-RNN-V model.

For the learning of the recurrent weights, we compare three learning methods inspired by PC. All three models share the same architecture, represented in Figure 3. On top of the top-down computations predicting the output ***x***_*t*_, these models include bottom-up computations updating the value of the hidden state, and providing a prediction error signal on the hidden layer:


(10) $$\begin{array}{rc} \epsilon_{x,t} & =x_{t}^{*}-x_{t} \end{array}$$

(11) $$\begin{array}{rc} h_{t}^{*} & =h_{t}+\alpha_{x}W_{b}\cdot \epsilon_{x,t} \end{array}$$

(12) $$\begin{array}{rc} \epsilon_{h,t} & =h_{t}^{*}-h_{t} \end{array}$$

where α_*x*_ is an update rate that weights the importance of bottom-up information for the estimation of ***h***_*t*_.

In fact, the three models we compare propose the same update rule for the recurrent weights, they will only differ in their definition of the feedback weights, which impacts the recurrent weights update. The learning rule for the recurrent weights is based on the hidden state at time *t* and the prediction error on the hidden state layer at time *t*+1, according to the following equation:


(13) $$\begin{array}{r} W_{r}\leftarrow W_{r}+\lambda_{r}\epsilon_{h,t+1}\cdot \tanh{\left(h_{t}^{*}\right)}^{⊺} \end{array}$$

where λ_*r*_ is the learning rate of the recurrent weights.

The difference between the three models lies in the computation of $h_{t+1}^{*}$. In the first model, that we label PC-RNN-V (for Vanilla), this bottom-up computation is done using the transposed of the top-down weights used for prediction. This results in a direct minimization of VFE, as shown in Appendix A. In the two other models, these feedback and bottom-up weights are instead learned. In the original PC model described in Rao and Ballard (1997), it was proposed to learn these feedback weights using the same rule as Equation 3 (up to a transpose to match the feedback weights shape):


(14) $$\begin{array}{r} W_{b}\leftarrow W_{b}+\lambda \tanh\left(h_{t}\right)\cdot \epsilon_{x,t}^{⊺} \end{array}$$

This learning rule ensures that with random initializations, but enough training time, the feedback weights converge to the transposed forward weights. Since this learning rule is a copy of the Hebbian rule used in Equation 3, we call PC-RNN-Hebb the RNN model using this method. The last model, inspired by the P-TNCN (Ororbia et al., 2020), implements a different learning rule for the feedback weights, described by the following equation:


(15) $$W_{b}\leftarrow \;W_{b}-{\text{λ}}_{b}\left(\epsilon_{h,t}-\;\epsilon_{h,t-1}\right)\;\cdot \;\epsilon_{x,t}^{⊺}$$

The model presented in Ororbia et al. (2020) also implements an additional term in the learning rule for the recurrent and output weights, on top of the rules explained here. This additional term led our experiments to worse results. For this reason, we do not provide more details about this rule and turn it off during the experiments shown below.

#### 3.2.3. Input Weights

**Figure 4:**
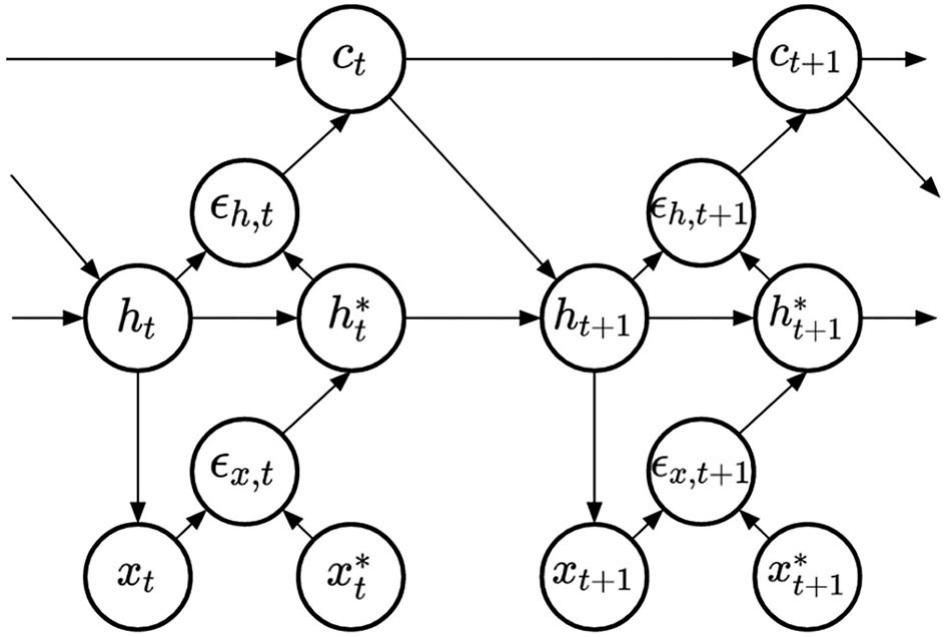
PC-RNN-HC model.

Finally, we compare four methods to learn RNN input weights. All methods share the same representation, displayed in Figure 4. This architecture was derived following the principle of free-energy minimization (Friston and Kilner, 2006), using a generative model that features a latent variable called hidden causes and labeled ***c***. Similarly to hidden states, hidden causes are hidden variables that can be dynamically inferred by the PC-RNN network. However, contrary to the hidden state variable, hidden causes are not dynamic: in the absence of prediction error the value of the hidden causes is stationary ***c***_*t*_ = ***c***_0_. The derivations of these models are summarized in Appendix A. The resulting architecture takes as input an initial value for the hidden causes and predicts an output sequence while dynamically updating the hidden states and hidden causes. During training, this input is the one-hot encoded index of the current task ***c***_0_ = *k*.

The four models differ according to two dimensions: whether they use evolution strategies to estimate the input weights, and according to the implementation of the influence of the input onto the hidden state dynamics. This influence can be either additive or multiplicative, the additive scheme is based on the following equation:


(16) $$h_{t}=(1-\frac{1}{\tau })h_{t-1}^{*}+\frac{1}{\tau }(W_{r}\cdot \tanh(h_{t-1}^{*})+W_{i}\cdot c_{t-1})$$

The multiplicative scheme is based on the following equation:


(17) $$\begin{array}{l} h_{t}=(1-\frac{1}{\tau })h_{t-1}^{*}\\ \;+\frac{1}{\tau }W_{f}^{⊺}\cdot ((W_{p}\cdot \tanh(h_{t-1}^{*}))⊙(W_{i}\cdot c_{t-1})) \end{array}$$

where we have introduced new synaptic weights ***W***_*p*_ and ***W***_*f*_, that replace the recurrent weights of the additive version. This reparameterization is used to reduce the total number of parameters of the multiplicative RNN, as already used in Annabi et al. (2021a,b).

We label these two models, respectively, PC-RNN-HC-A and PC-RNN-HC-M, the HC suffix standing for Hidden Causes and the A and M suffixes standing for Additive and Multiplicative. The differences between the additive and multiplicative models also impact the bottom-up update rule for ***c***_*t*_. However, in our experiments, we always turn off this mechanism by using an update rate equal to zero.

In these two first methods, the learning rules for the input weights follow the PC theory and attempt at minimizing the prediction error on the hidden layer. The learning rule used for the PC-RNN-HC-A model is the following:


(18) $$\begin{array}{r} W_{i}\leftarrow W_{i}+\lambda_{i}\epsilon_{h,t+1}\cdot c_{t}^{⊺} \end{array}$$

For the PC-RNN-HC-M model, we obtain the following rule:


(19) $$W_{i}\leftarrow W_{i}+{\text{λ}}_{i}((W_{p}\cdot \tanh(h_{t}^{*}))⊙(W_{f}\cdot \;\epsilon_{h,t+1}))\cdot c_{t}^{⊺}$$

The third and fourth methods that we study are respectively based on the PC-RNN-HC-A and PC-RNN-HC-M, but instead use random search to optimize the weights ***W***_*i*_. Our implementation of this random search is inspired by the learning algorithm proposed in Pitti et al. (2017):


(20) $$\begin{array}{r} \delta_{i}\sim N\left(0,\sigma^{2}I_{d_{h}^{2}}\right) \end{array}$$

(21) $$\begin{array}{r} ||\epsilon_{x,i}|{|}_{2}\leftarrow \text{simulate}\left(W_{i}+\delta_{i}\right) \end{array}$$

(22) $$\begin{array}{r} W_{i}\leftarrow W_{i}+\delta_{i}sign\left(||\epsilon_{x,i-1}|{|}_{2}-||\epsilon_{x,i}|{|}_{2}\right) \end{array}$$

where the function *sign* associates −1 to negative values and 1 to positive values. At each training iteration *i*, the algorithm samples a noise matrix ***δ***_*i*_ that is added to the input weights of the RNN. After generation, the difference between the old and new average norm of the prediction error ||***ϵ***_*x, i*−1_||_2_−||***ϵ***_*x, i*_||_2_ is used as a measure of success of the addition of ***δ***_*i*_ and weights the update of ***W***_*i*_. Since this algorithm only relies on an average of the prediction error over the predicted sequences, that can be computed iteratively, it qualifies as an online learning algorithm.

In summary, we have identified four learning algorithms for output weights, three learning algorithms for recurrent weights, and four learning algorithms for input weights. To connect the proposed methods to the classification of continual learning methods presented above, we could categorize the Conceptors method as a regularization method, and the fact that new tasks are associated with new inputs to the RNN in the shape of hidden causes, as an architecture modification method.

## 4. Results

### 4.1. Hyperparameter Optimization

The source code for the experiments presented in this section is available on GitHub^1^. It contains our implementation of the different models as well as the hyperparameter optimization method. In Appendix B, we provide the optimal hyperparameters found for each model.

**Figure 5:**
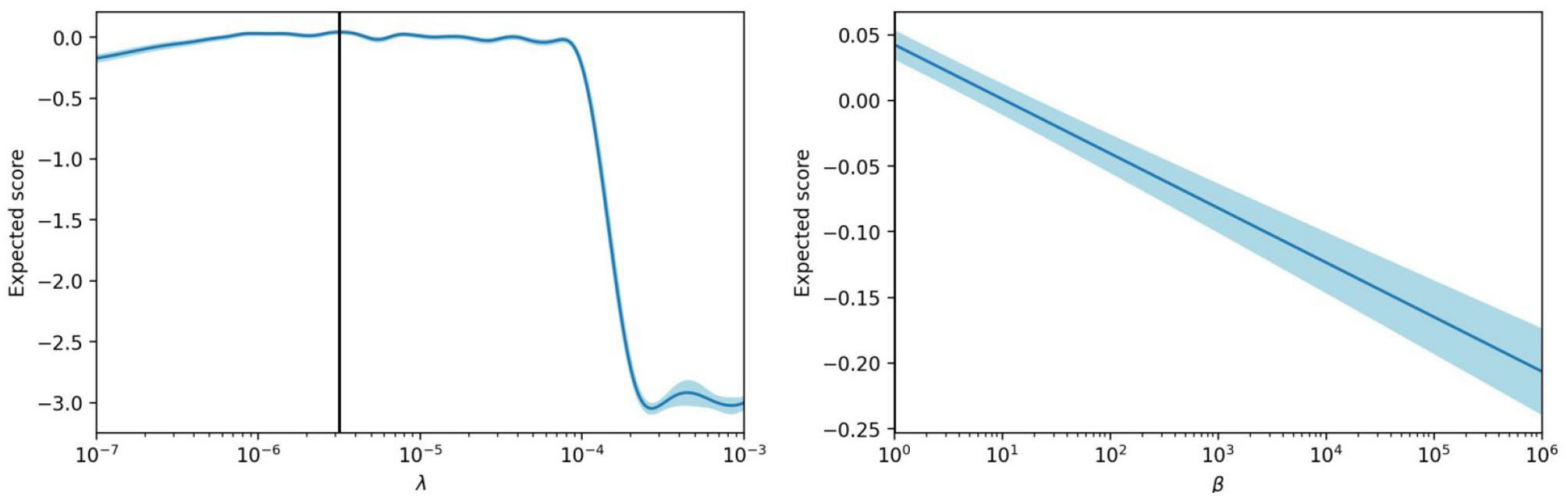
Score estimation of the hyperparameter optimizer with regard to the learning rate λ and the coefficient β, for the EWC model.

We start by showing an example of a hyperparameter optimization in Figure 5, which was performed on the EWC model with *d*_*h*_ = 300. The optimized hyperparameters are the learning rate of the output weights, λ, and the coefficient β. After trying 200 hyperparameter configurations, the optimizer can estimate the score for all the configurations within the given range of values. These figures display the evolution of the score estimation according to λ using the optimal value for β, and according to β using the optimal value for λ. We can see that the function according to β monotonically decreases, while the function according to λ increases steadily before dropping once we attain values of the learning rate that no longer sustain convergence of the gradient descent.

In this case, the hyperparameter optimization has found that the EWC regularization does not improve the final score, and suggests using the lowest possible value for the coefficient β. When β increases, the regularization mitigates catastrophic forgetting but prevents proper learning of new tasks.

For all the results presented below, we perform optimization of the hyperparameters following the same protocol.

### 4.2. Output Weights

**Figure 6:**
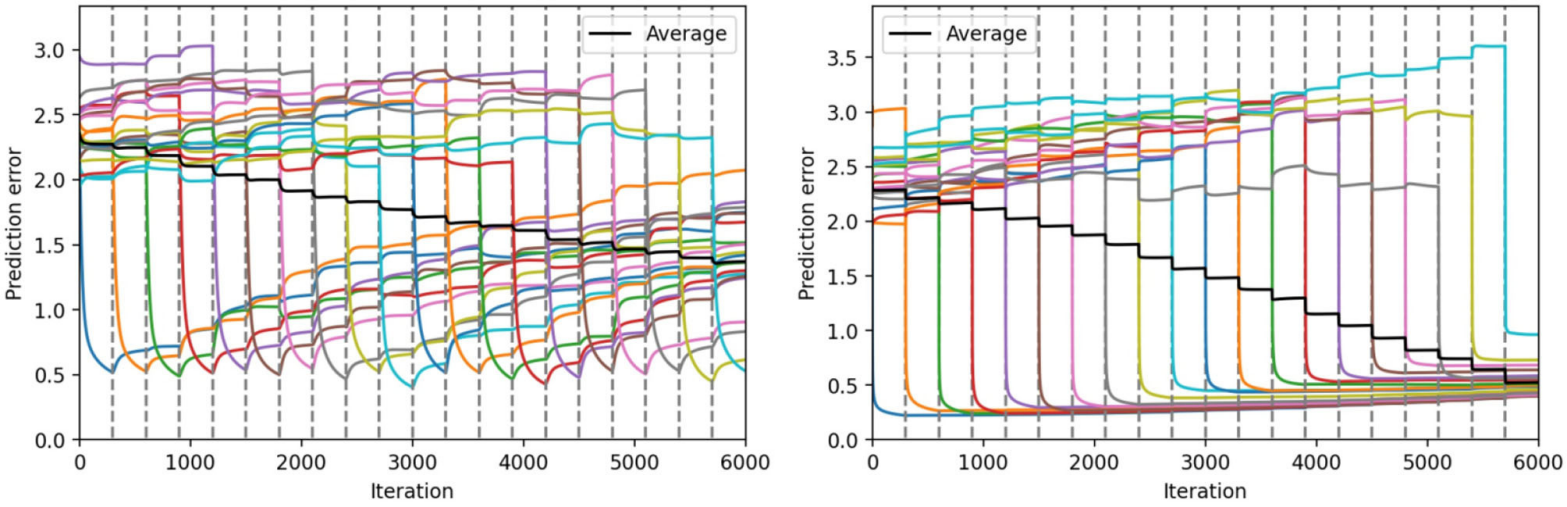
Continual learning results with the ESN model (left) and the Conceptors model (right). We represent the average prediction error over 10 seeds, for the continual learning of 20 sequential patterns, obtained on the first test set. The colored lines correspond to the prediction error on each individual task, and the black line corresponds to the prediction error averaged on all tasks. The 20 tasks are delimited by the dashed gray lines.

In Figure 6, we represent the average prediction error over 10 seeds for the continual learning of 20 sequential patterns obtained on the test set, with the hyperparameters found using the protocol described before. The vertical dashed lines in these figures delimit each of the training tasks. The colored lines represent the individual prediction error for each of the 20 sequence patterns (averaged over the 10 seeds). Finally, the black line represents the average prediction error over all the sequence patterns (averaged over the 10 seeds).

During each task (for each colored line), we can observe that one of the individual prediction errors decreases rapidly, while the other prediction errors only slightly change. Once the training task corresponding to a certain sequence pattern *k* is over, the prediction error associated with this pattern tends to increase. The better learning mechanism is the one that can limit this undesirable forgetting of previously learned sequence patterns. We can observe in Figure 6 the Conceptors learning mechanism limits forgetting compared to the standard stochastic gradient descent rule used in our ESN model.

At first, it can be surprising that for each individual task, the corresponding prediction error reaches a lower value for the Conceptors model than for the ESN model. In terms of learning rules, the ESN model could potentially learn each pattern with better accuracy by increasing the learning rate. However, the hyperparameter optimizer has estimated that an increased learning rate would be detrimental to the complete continual learning task. Indeed, increasing the learning rate might improve the learning on every individual task, but it would also lead to more forgetting throughout the complete task. It is only because the Conceptors learning mechanisms naturally limit forgetting that the hyperparameter optimized “allows” a higher learning rate and, thus, better learning on each individual task.

We can also observe that the prediction error level that is reached during each individual task using the Conceptors model seems to increase throughout the complete task. We suppose that this is a consequence of further learning being prevented on synaptic connections associated with previous tasks' associated Conceptors. When a large number of individual tasks are over, learning is limited to synapses corresponding to a subspace of the hidden state space not belonging to any of the previous Conceptors. Decreasing the aperture α would allow better learning of the late tasks, but at the detriment of an increased forgetting of the early tasks.

**Figure 7:**
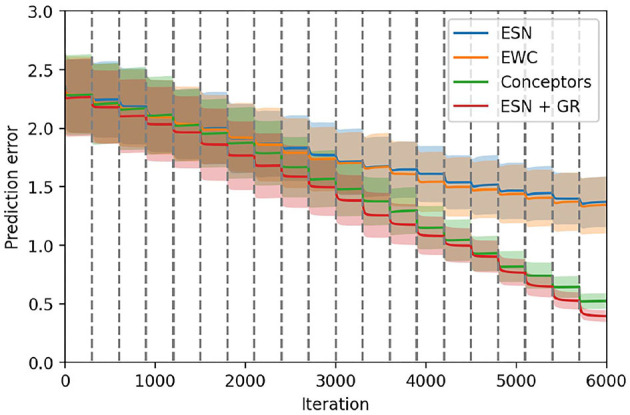
Comparison between the four learning methods for the output weights on the first test set. The 20 tasks are delimited by the dashed gray lines.

Figure 7 compiles these previous figures to compare the average prediction error using the four learning mechanisms for output weights. At the end of the training, we can see that the Conceptors model and generative replay achieve a significantly lower prediction error than the ESN using the standard stochastic gradient descent rule and the EWC regularization for the learning of the output weights.

As explained in the last section, the hyperparameters found for EWC correspond to a configuration where the regularization is almost removed, and the EWC model, thus, has the same performance as the ESN model.

The generative replay strategy outperforms all other approaches, but at the cost of a longer training time. Indeed, at each task *k*, the model is trained on (*k*−1) replayed trajectories on top of the current trajectory. For all models, we have limited the number of training iterations on each task, which induces an unfair advantage for generative replay in our experiments. For this reason, we do not include this technique in the remaining comparisons.

The results obtained with these models on the three data sets (validation set and two test sets) are provided in Table 2, together with the results for the learning of recurrent and input weights, discussed in the next sections.

**Table 2:** Average prediction error after training on all *p* tasks.

	**Validation**	**Test 1**	**Test 2**
**Model**	**(MOCAP**,	**(MOCAP**,	**(Handwriting**,
	***p* = 15)**	***p* = 20)**	***p* = 20)**
ESN	0.90 ± 0.07	1.37 ± 0.14	0.71 ± 0.04
EWC	0.90 ± 0.09	1.35 ± 0.15	0.69 ± 0.05
Conceptors	0.31 ± 0.02	0.52 ± 0.04	0.27 ± 0.02
ESN + GR	**0.29 ± 0.01**	**0.39 ± 0.01**	**0.22 ± 0.01**
PC-RNN-V	0.87 ± 0.09	1.41 ± 0.14	0.79 ± 0.10
P-TNCN	0.90 ± 0.08	1.42 ± 0.18	0.71 ± 0.05
PC-RNN-Hebb	0.90 ± 0.07	1.41 ± 0.10	0.73 ± 0.05
PC-RNN-HC-A	**0.74 ± 0.09**	**1.28 ± 0.22**	**0.59 ± 0.04**
PC-RNN-HC-M	0.81 ± 0.04	1.32 ± 0.09	0.77 ± 0.05
PC-RNN-HC-A-RS	0.90 ± 0.08	1.39 ± 0.15	0.77 ± 0.05
PC-RNN-HC-M-RS	0.93 ± 0.06	1.38 ± 0.10	0.72 ± 0.05
PC-Conceptors	**0.28 ± 0.01**	**0.36 ± 0.02**	**0.18 ± 0.01**

*Bold value indicates the best performance in each group of models*.

### 4.3. Recurrent Weights

In this second experiment, we compare the PC-RNN-V with two variants using learning rules for the feedback weights instead of using the transposed feedforward weights. These three learning methods in the end provide different update rules for the recurrent weights of the RNN. The results of this second comparative analysis are provided in Table 2.

We can see that none of the three models brings any significant improvement compared with the ESN, which is exactly the same model without any learning occurring on the recurrent weights. In terms of hyperparameters, only the PC-RNN-V has an optimal learning rate for recurrent weights that does not correspond to the lowest value authorized during hyperparameter optimization. This means that for both P-TNCN and PC-RNN-Hebb models, the hyperparameter optimizer has estimated that training the recurrent weights only hinders the final prediction error. For the PC-RNN-V model, a slight improvement was found in the validation set using the learning rule for recurrent weights, but this improvement does not transfer to the two test sets.

We can conclude based on these results that recurrent weights learning in a continual learning setting is difficult and might often lead to more catastrophic forgetting.

### 4.4. Input Weights

**Figure 8:**
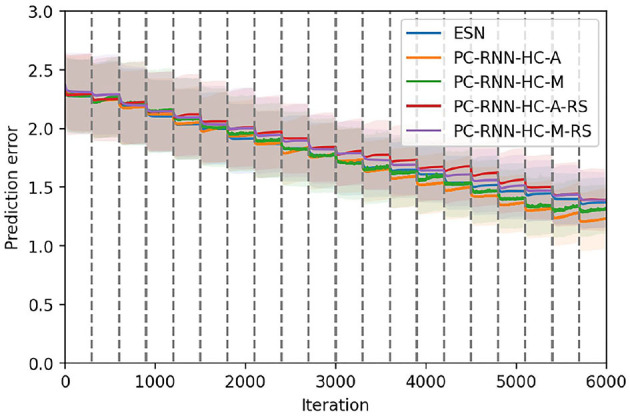
Comparison between the three learning methods for the input weights. The PC-RNN-V model, where no learning is performed on the input weights, is also displayed as a baseline. The 20 tasks are delimited by the dashed gray lines.

Figure 8 displays the results obtained with the four learning mechanisms for input weights, and the ESN as a baseline. We use the ESN as a baseline to measure the improvement brought by the learning in the input layer. The results of the validation set and other test sets are displayed in Table 2.

These results suggest that the learning methods using random search (RS suffix) perform poorly compared to the corresponding learning rules relying on the propagation of error using PC. The two models using random search perform similarly to the baseline ESN model. This observation is surprising since the ***W***_*i*_ weights in PC-RNN-HC-A/M architectures are directly factored according to each individual task. Indeed, during the task *k*, we can limit learning on the *k*-th column of the ***W***_*i*_ weights, since these are the only weights that influence the RNN trajectory. Consequently, training this layer should not cause any additional forgetting, and thus should only bring improvements over the baseline ESN model. Since the two models using random search did not bring any improvement, we suppose that this is due to the limited number of iterations allowed for the training on each individual task. We observed that in general training with random search as in the INFERNO model (Pitti et al., 2017) needed many more iterations than gradient-based methods.

The PC-RNN-HC-A/M models trained using the PC-based learning rules still showed some significant improvement compared with the ESN baseline, with the PC-RNN-HC-A model performing slightly better than the PC-RNN-HC-M model. This experiment allows us to conclude that the learning rule for input weights proposed by the PC-RNN-HC-A model is the most suited to a continual learning setting.

### 4.5. Combining Conceptors and Hidden Causes

**Figure 9:**
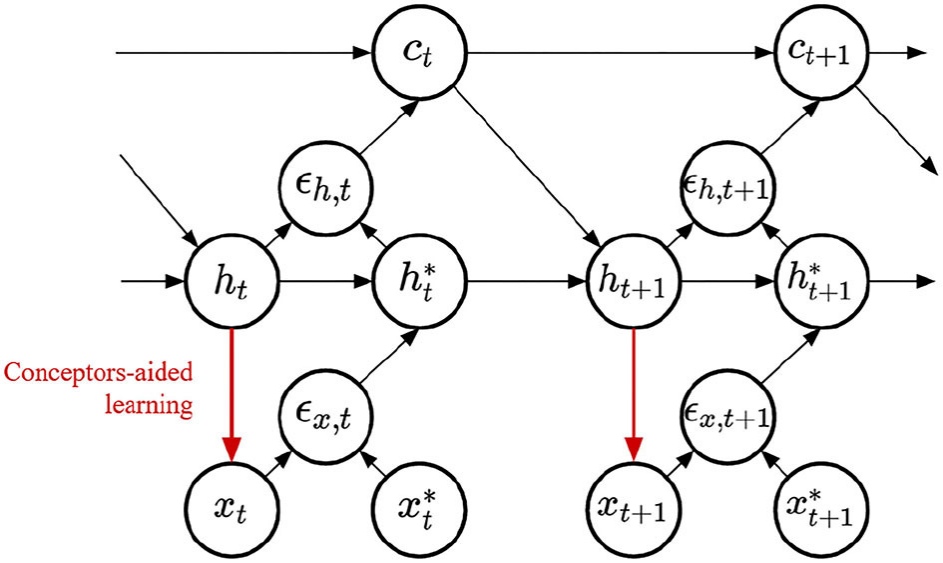
PC-Conceptors model.

**Figure 10:**
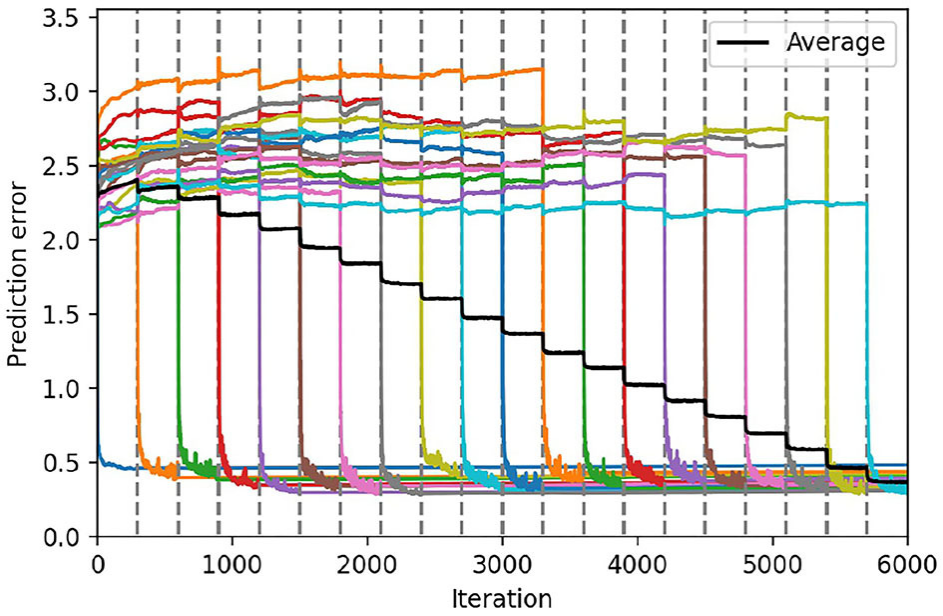
Continual learning results using the PC-Conceptors. We represent the average prediction error over 10 seeds, for the continual learning of 20 sequential patterns, using the PC-RNN-HC-A model with Conceptors. The colored lines correspond to the prediction error on each individual task, and the black line corresponds to the prediction error averaged on all tasks. The 20 tasks are delimited by the dashed gray lines.

Finally, we can inquire whether these different learning mechanisms combine well with each other. We implement the Conceptors learning rule on the output weights of a PC-RNN-HC-A model, a new model that we label PC-Conceptors, as represented in Figure 9. Figure 10 displays the prediction error on each individual task as well as the average prediction error throughout learning, using this model. Interestingly, virtually no forgetting seems to happen during learning, as the individual prediction errors plateau after decreasing during the corresponding individual tasks.

Additionally, the hyperparameter optimizer in this case recommended using the lowest possible value for the recurrent weights learning rate. This suggests that the recurrent weights learning negatively interferes with the Conceptors model. The Conceptors model might be sensible for recurrent weight learning, since this could turn the previously learned Conceptors into obsolete descriptors of the corresponding hidden state trajectories.

**Figure 11:**
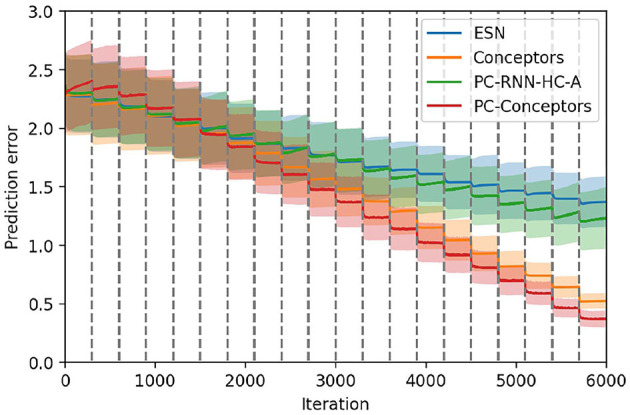
Comparison between the ESN, the Conceptors model, the PC-RNN-HC-A model, and the PC-Conceptors model on the first test set. The 20 tasks are delimited by the dashed gray lines.

We compare these results with the ESN, Conceptors and PC-RNN-HC-A models in Figure 11, which confirms that this combination of learning methods seems to provide the RNN model best suited for online continual learning.

## 5. Discussion

Overall, this study suggests that regularization methods such as Conceptors, and architectural methods, as proposed in the PC-RNN-HC architectures, can help design RNN models with online learning rules suitable for continual learning.

Additionally, we have found that combining Conceptors-based learning for the output weights with PC-based learning for the input weights further improves the model precision. In future study, it would be interesting to investigate whether the combination of these two mechanisms could be improved. Especially, the learning of the input weights is only driven by the minimization of the prediction error on the recurrent layer. This could be improved by integrating an orthogonality criterion to the learning rule: if the input weights are optimized in order to decorrelate the different hidden state trajectories, it could facilitate the learning of the output weights.

The models we have proposed also suffer from another limitation that should be addressed in future work. The models were trained using as input the current task index, which is information that might not be available in realistic lifelong learning settings. The model should be able to detect a distributional shift when it occurs and adapt its learning rules based on these events.

## Data Availability Statement

The datasets presented in this study can be found in online repositories. The name of the repository and accession number can be found below: GitHub, https://github.com/sino7/continual_online_learning_rnn_benchmark.

## Author Contributions

The models and experiments were designed by LA, AP, and MQ. The models and experiments were implemented by LA. The article was written by LA with instructions and feedback from AP and MQ. All authors contributed to the article and approved the submitted version.

## Funding

This study was funded by the Cergy-Paris University Foundation (Facebook grant) and partially by Labex MME-DII, France (ANR11-LBX-0023-01).

## Conflict of Interest

The authors declare that the research was conducted in the absence of any commercial or financial relationships that could be construed as a potential conflict of interest.

## Publisher's Note

All claims expressed in this article are solely those of the authors and do not necessarily represent those of their affiliated organizations, or those of the publisher, the editors and the reviewers. Any product that may be evaluated in this article, or claim that may be made by its manufacturer, is not guaranteed or endorsed by the publisher.

## Footnotes

^1^ https://github.com/sino7/continual_online_learning_rnn_benchmark
