# Supplementary material for: Continual Sequence Modeling With Predictive Coding
Source: Front Neurorobot. 2022 May 23;16:845955. doi: 10.3389/fnbot.2022.845955 (PMC9171436; doi:10.3389/fnbot.2022.845955)
Supplement: Supplementary file 1 [file Data_Sheet_1.pdf]

Appendices

A. PC-RNN models

The Free Energy Principle (FEP) (Friston, 2009, 2010) is a framework aiming at unifying cognition under the common principle of surprise minimization. Starting from the FEP hypotheses and certain generative models, and relying on a few simplifications, we derive an algorithm that can be translated as neural network dynamics aligning with the PC theory. We present here the derivations for the PC-RNN-V and the PC-RNN-HC models used in the main text.

For a more detailed understanding of the link between PC and the FEP, we suggest looking at the very useful mathematical reviews presented in (Bogacz, 2017) and (Buckley et al., 2017).

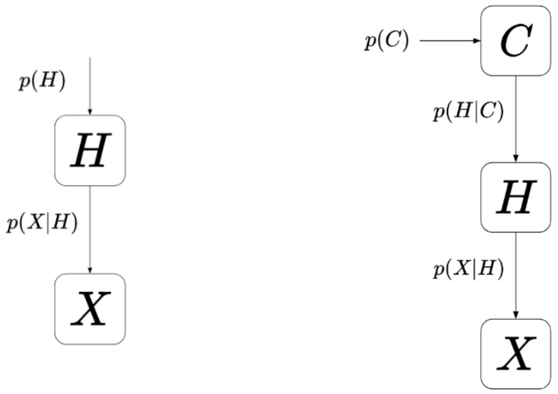

Figure 12: Probabilistic graphical models of the PC-RNN-V model (left) and of the PC-RNN-HC model (right).

A.1. PC-RNN-V

The PC-RNN-V model is based on a simple probabilistic generative model, represented on the left in figure 12. The generative model is composed of two multivariate random variables $\boldsymbol{H}$, of dimension d_h, and $\boldsymbol{X}$, of dimension d_x, related according to the following probability distributions:

$$p(\boldsymbol{h}) = \mathcal{N}(\boldsymbol{h}; \boldsymbol{\mu}_h, \sigma_h^2 \mathbb{I}_{d_h}) \tag{23}$$

$$p(\boldsymbol{x}|\boldsymbol{h}) = \mathcal{N}(\boldsymbol{x}; \boldsymbol{g}(\boldsymbol{h}), \sigma_x^2 \mathbb{I}_{d_x}) \tag{24}$$

The prior density $p(\boldsymbol{h})$ and the likelihood density $p(\boldsymbol{x}|\boldsymbol{h})$ are both assumed to be multivariate Gaussians. $\boldsymbol{\mu}_h$ and $\sigma_h^2 \mathbb{I}_{d_h}$ denote the prior density mean and covariance matrix, and $\boldsymbol{g}(\boldsymbol{h})$ and $\sigma_x^2 \mathbb{I}_{d_x}$ denote the likelihood density mean and covariance matrix. We assume that the covariance matrices are proportional to the identity matrices of dimension d_h and d_x.

The function $\boldsymbol{g}$ performs a linear mapping from the variable $\boldsymbol{H}$ on which we first apply the hyperbolic tangent function:

$$\boldsymbol{g}(\boldsymbol{h}) = \boldsymbol{W}_o \cdot \tanh(\boldsymbol{h}) \tag{25}$$

where $\boldsymbol{W}_o$ is a matrix of dimension (d_x, d_h), corresponding to the output weights of our RNN model. To perform variational inference, we introduce a recognition density function $q(\boldsymbol{H})$:

$$q(\boldsymbol{h}) = \mathcal{N}(\boldsymbol{h}; \boldsymbol{m}_h, v_h \mathbb{I}_{d_h}) \tag{26}$$

This recognition density is also assumed to be Gaussian, with mean $\boldsymbol{m}_h$ and variance v_h. Using these definitions, we can derive a simplified expression of the variational free-energy (VFE):

$$
\begin{aligned}
F(\boldsymbol{x}^*, \boldsymbol{m}_h) = & \frac{1}{2\sigma_x^2}\|\boldsymbol{x}^* - \boldsymbol{g}(\boldsymbol{m}_h)\|_2^2 + \frac{d_x}{2}\log(\sigma_x^2) \\
& + \frac{1}{2\sigma_h^2}\|\boldsymbol{m}_h - \boldsymbol{\mu}_h\|_2^2 + \frac{d_h}{2}\log(\sigma_h^2) \\
& + C
\end{aligned}
\tag{27}
$$

We can now derive from this expression the gradient of the VFE with regard to the recognition density parameter $\boldsymbol{m}_h$, which results in the following gradient descent update rule for $\boldsymbol{m}_h$:

$$
\begin{aligned}
\boldsymbol{m}_h \leftarrow \boldsymbol{m}_h & + \frac{\alpha}{\sigma_x^2}\big(1 - \tanh^2(\boldsymbol{m}_h)\big) \odot \big(\boldsymbol{W}_o^\intercal \cdot \boldsymbol{\epsilon}_x\big) \\
& - \frac{\alpha}{\sigma_h^2}\boldsymbol{\epsilon}_h
\end{aligned}
\tag{28}
$$

In this equation, we have introduced the notations $\boldsymbol{\epsilon}_x = \boldsymbol{x}^* - \boldsymbol{g}(\boldsymbol{m}_h)$ and $\boldsymbol{\epsilon}_h = \boldsymbol{m}_h - \boldsymbol{\mu}_h$ that correspond to the prediction errors at different layers of the generative model. Finally, to make this model able to generate temporal patterns, we make the prior density mean $\boldsymbol{\mu}_h$ dependent on the value of the recognition density mean $\boldsymbol{m}_h$:

$$
\boldsymbol{\mu}_h = (1 - \frac{1}{\tau})\boldsymbol{m}_h + \frac{1}{\tau}\boldsymbol{W}_r \cdot \tanh\big(\boldsymbol{m}_h\big) \tag{29}
$$

where τ and $\boldsymbol{W}_r$ respectively correspond to the time constant and the recurrent weights of our RNN. Using equations 28 and 29, we can construct a system of equations guiding the temporal evolutions of the variables of our neural network:

$$
\boldsymbol{\mu}_{h,t} = (1 - \frac{1}{\tau})\boldsymbol{m}_{h,t-1}^{post} + \frac{1}{\tau}\boldsymbol{W}_r \cdot \tanh\big(\boldsymbol{m}_{h,t-1}^{post}\big) \tag{30}
$$

$$
\boldsymbol{\epsilon}_{h,t} = \boldsymbol{m}_{h,t-1}^{post} - \boldsymbol{\mu}_{h,t} \tag{31}
$$

$$
\boldsymbol{m}_{h,t}^{prior} = \boldsymbol{m}_{h,t-1}^{post} - \frac{\alpha}{\sigma_h^2}\boldsymbol{\epsilon}_{h,t} \tag{32}
$$

$$
\hat{\boldsymbol{x}}_t = \boldsymbol{W}_o \cdot \tanh\big(\boldsymbol{m}_{h,t}^{prior}\big) \tag{33}
$$

$$
\boldsymbol{\epsilon}_{x,t} = \boldsymbol{x}_t^* - \hat{\boldsymbol{x}}_t \tag{34}
$$

$$
\boldsymbol{m}_{h,t}^{post} = \boldsymbol{m}_{h,t}^{prior} + \frac{\alpha}{\sigma_x^2}\big(1 - \tanh^2(\boldsymbol{m}_{h,t}^{prior})\big) \odot \big(\boldsymbol{W}_o^\intercal \cdot \boldsymbol{\epsilon}_{x,t}\big) \tag{35}
$$

By merging the first three equations, and recombining the hyperparameters τ, α, σ_h and σ_x into the hyperparameters $\tau \leftarrow \frac{\tau\sigma_h^2}{\alpha}$ and $\alpha_x \leftarrow \frac{\alpha}{\sigma_x^2}$ we obtain the equations of the PC-RNN-V model as presented in the main text. The hidden state $\boldsymbol{h}_t$ of the PC-RNN-V corresponds to the recognition density mean $\boldsymbol{m}_{h,t}^{prior}$, while the updated hidden state $\boldsymbol{h}'_t$ corresponds to the updated (or posterior) recognition density mean $\boldsymbol{m}_{h,t}^{post}$.

The FEP also provides update rules for model parameters. If we also minimize VFE by a gradient descent on the parameters $\boldsymbol{W}_o$ and $\boldsymbol{W}_r$, we obtain the following learning rules:

$$
\boldsymbol{W}_o \leftarrow \boldsymbol{W}_o + \lambda\boldsymbol{\epsilon}_{x,t} \cdot \tanh(\boldsymbol{m}_{h,t}^{prior})^\intercal \tag{36}
$$

$$
\boldsymbol{W}_r \leftarrow \boldsymbol{W}_r + \lambda_r\boldsymbol{\epsilon}_{h,t+1} \cdot \tanh(\boldsymbol{m}_{h,t}^{post})^\intercal \tag{37}
$$

A.2. PC-RNN-HC

In the PC-RNN-HC model, we add a new multivariate random variable influencing the temporal dynamics of $\boldsymbol{H}$. To align with the FEP literature, we name this variable hidden causes, and denote it as $\boldsymbol{C}$. The

corresponding probabilistic model is represented on the right in figure 12. It is described by the following set of equations:

$$p(\boldsymbol{c}) = \mathcal{N}(\boldsymbol{c}; \boldsymbol{\mu}_c, \sigma_c^2 \mathbb{I}_{d_c}) \tag{38}$$

$$p(\boldsymbol{h}|\boldsymbol{c}) = \mathcal{N}(\boldsymbol{h}; \boldsymbol{f}(\boldsymbol{c}, \boldsymbol{h}_{past}), \sigma_h^2 \mathbb{I}_{d_h}) \tag{39}$$

$$p(\boldsymbol{x}|\boldsymbol{h}) = \mathcal{N}(x; \boldsymbol{g}(\boldsymbol{h}), \sigma_x^2 \mathbb{I}_{d_x}) \tag{40}$$

Similarly to the first presented model, we use a "trick" to enforce temporal dynamics onto the variable $\boldsymbol{H}$. While in the first model, the future value of $\boldsymbol{H}$ only depended on its past value, here it also depends on the hidden causes variable $\boldsymbol{C}$. To perform variational inference, we introduce the recognition density function $q(\boldsymbol{H}, \boldsymbol{C})$:

$$q(\boldsymbol{h}, \boldsymbol{c}) = q(\boldsymbol{h})q(\boldsymbol{c}) \tag{41}$$

$$q(\boldsymbol{h}) = \mathcal{N}(\boldsymbol{h}; \boldsymbol{m}_h, v_h \mathbb{I}_{d_h}) \tag{42}$$

$$q(\boldsymbol{c}) = \mathcal{N}(\boldsymbol{c}; \boldsymbol{m}_c, v_c \mathbb{I}_{d_c}) \tag{43}$$

H and C are assumed to be independent according to this recognition density, which thus factors into two marginal density functions $q(\boldsymbol{H})$ and $q(\boldsymbol{C})$. Both marginal distributions are assumed to be Gaussian, respectively with means $\boldsymbol{m}_h$ and $\boldsymbol{m}_c$, and variances v_h and v_c. We can express the VFE as:

$$\begin{aligned} F(\boldsymbol{x}^*, \boldsymbol{m}_h, \boldsymbol{m}_c) = &\frac{1}{2\sigma_x^2}\|\boldsymbol{x}^* - \boldsymbol{g}(\boldsymbol{m}_h)\|_2^2 + \frac{d_x}{2}\log(\sigma_x^2) \\ &+ \frac{1}{2\sigma_h^2}\|\boldsymbol{m}_h - \boldsymbol{f}(\boldsymbol{m}_c, \boldsymbol{h}_{past})\|_2^2 + \frac{d_h}{2}\log(\sigma_h^2) \\ &+ \frac{1}{2\sigma_c^2}\|\boldsymbol{m}_c - \boldsymbol{\mu}_c\|_2^2 + \frac{d_c}{2}\log(\sigma_c^2) \\ &+ C \end{aligned} \tag{44}$$

We can derive from this expression the gradient of the VFE with regard to the recognition distribution parameters $\boldsymbol{m}_h$ and $\boldsymbol{m}_c$, which are used in the following update rules:

$$\begin{aligned} \boldsymbol{m}_h \leftarrow \boldsymbol{m}_h &+ \frac{\alpha}{\sigma_x^2}\big(1 - \tanh^2(\boldsymbol{m}_h)\big) \odot \big(\boldsymbol{W}_o^\intercal \cdot \boldsymbol{\epsilon}_x\big) \\ &- \frac{\alpha}{\sigma_h^2}\big(\boldsymbol{m}_h - \boldsymbol{f}(\boldsymbol{m}_c, \boldsymbol{m}_h)\big) \end{aligned} \tag{45}$$

$$\boldsymbol{m}_c \leftarrow \boldsymbol{m}_c \frac{\alpha}{\sigma_h^2}\big(\boldsymbol{m}_h - \boldsymbol{f}(\boldsymbol{m}_c, \boldsymbol{m}_h)\big) \cdot \nabla_{\boldsymbol{m}_c}\boldsymbol{f}(\boldsymbol{m}_c, \boldsymbol{m}_h) \tag{46}$$

These two update rules depend on the function $\boldsymbol{f}$ that we still have not defined. We consider two possible implementations. The first possibility is to use the additional variable $\boldsymbol{C}$ as an additive influence on the temporal dynamics of $\boldsymbol{H}$, while the second possibility considers a multiplicative influence on the temporal dynamics of $\boldsymbol{H}$.

The additive hidden causes computational model can be described with the following set of equations:

$$\boldsymbol{m}_{h,t}^{prior} = (1 - \frac{\alpha}{\tau\sigma_h^2})\boldsymbol{m}_{h,t-1}^{post} + \frac{\alpha}{\tau\sigma_h^2}\big(\boldsymbol{W}_r \cdot \tanh(\boldsymbol{m}_{h,t-1}^{post}) + \boldsymbol{W}_i \cdot \boldsymbol{m}_{c,t-1}\big) \tag{47}$$

$$\hat{\boldsymbol{x}}_t = \boldsymbol{W}_o \cdot \tanh(\boldsymbol{m}_{h,t}^{prior}) \tag{48}$$

$$\boldsymbol{\epsilon}_{x,t} = \boldsymbol{x}_t^* - \hat{\boldsymbol{x}}_t \tag{49}$$

$$\boldsymbol{m}_{h,t}^{post} = \boldsymbol{m}_{h,t}^{prior} + \frac{\alpha}{\sigma_x^2}\big(1 - \tanh^2(\boldsymbol{m}_{h,t}^{prior})\big) \odot \big(\boldsymbol{W}_o^\intercal \cdot \boldsymbol{\epsilon}_{x,t}\big) \tag{50}$$

$$\boldsymbol{\epsilon}_{h,t} = \boldsymbol{m}_{h,t}^{post} - \boldsymbol{m}_{h,t}^{prior} \tag{51}$$

$$\boldsymbol{m}_{c,t} = \boldsymbol{m}_{c,t-1} + \frac{\alpha}{\tau\sigma_h^2}\boldsymbol{W}_i^\intercal \cdot \boldsymbol{\epsilon}_{h,t} \tag{52}$$

The multiplicative hidden causes model is described by the same equations, except for equations 47 and 52 that are respectively replaced by:

$$\boldsymbol{m}_{h,t}^{prior} = (1 - \frac{\alpha}{\tau\sigma_h^2})\boldsymbol{m}_{h,t-1}^{post} + \frac{\alpha}{\tau\sigma_h^2}\boldsymbol{W}_f^\intercal \cdot \Big(\big(\boldsymbol{W}_p \cdot \tanh(\boldsymbol{m}_{h,t-1}^{post})\big) \odot (\boldsymbol{W}_i \cdot \boldsymbol{m}_{c,t-1})\Big) \tag{53}$$

$$\boldsymbol{m}_{c,t} = \boldsymbol{m}_{c,t-1} + \frac{\alpha}{\tau\sigma_h^2}\Big(\big(\boldsymbol{W}_p \cdot \tanh(\boldsymbol{m}_{h,t-1}^{post})\big) \odot \boldsymbol{W}_i\Big)^\intercal \cdot \boldsymbol{W}_f \cdot \boldsymbol{\epsilon}_{h,t} \tag{54}$$

The hidden states $\boldsymbol{h}_t$ and $\boldsymbol{h}'_t$, and hidden causes $\boldsymbol{c}_t$ of the PC-RNN-HC correspond to the recognition density means $\boldsymbol{m}_{h,t}^{prior}$, $\boldsymbol{m}_{h,t}^{post}$, and $\boldsymbol{m}_{c,t}$.

The FEP also provides update rules for model parameters. If we also minimize VFE by a gradient descent on the parameters synaptic weights, we obtain the following learning rules, starting with the output weights:

$$\boldsymbol{W}_o \leftarrow \boldsymbol{W}_o + \frac{\lambda}{\sigma_x^2}\boldsymbol{\epsilon}_{x,t} \cdot \tanh(\boldsymbol{m}_{h,t}^{prior})^\intercal \tag{55}$$

where λ is the learning rate associated with the weight matrix $\boldsymbol{W}_o$. This rule locally minimizes VFE by updating the model parameters in a direction that minimize the local prediction error $\boldsymbol{\epsilon}_x$. We can apply the same method to derive the learning rules on the weights $\boldsymbol{W}_r$ and $\boldsymbol{W}_i$ in the additive model:

$$\boldsymbol{W}_r \leftarrow \boldsymbol{W}_r + \frac{\lambda_r}{\tau\sigma_h^2}\boldsymbol{\epsilon}_{h,t+1} \cdot \tanh(\boldsymbol{m}_{h,t}^{post})^\intercal \tag{56}$$

$$\boldsymbol{W}_i \leftarrow \boldsymbol{W}_i + \frac{\lambda_c}{\tau\sigma_h^2}\boldsymbol{\epsilon}_{h,t+1} \cdot \boldsymbol{m}_{c,t}^\intercal \tag{57}$$

where λ_r and λ_c are the learning rates associated with the weight matrices $\boldsymbol{W}_r$ and $\boldsymbol{W}_i$. For the multiplicative model, the local gradient descent derivations are a bit more complex, and provide the following learning rules for the $\boldsymbol{W}_p$, $\boldsymbol{W}_f$ and $\boldsymbol{W}_i$:

$$\boldsymbol{W}_p \leftarrow \boldsymbol{W}_p + \frac{\lambda_r}{\tau\sigma_h^2}\tanh(\boldsymbol{m}_{h,t}^{post}) \cdot \big((\boldsymbol{W}_i \cdot \boldsymbol{m}_{c,t}) \odot (\boldsymbol{W}_f \cdot \boldsymbol{\epsilon}_{h,t+1})\big)^\intercal \tag{58}$$

$$\boldsymbol{W}_f \leftarrow \boldsymbol{W}_f + \frac{\lambda_r}{\tau\sigma_h^2}\boldsymbol{\epsilon}_{h,t+1} \cdot \Big(\big(\boldsymbol{W}_i \cdot \boldsymbol{m}_{c,t}\big) \odot \big(\boldsymbol{W}_p \cdot \tanh(\boldsymbol{m}_{h,t}^{post})\big)\Big)^\intercal \tag{59}$$

$$\boldsymbol{W}_i \leftarrow \boldsymbol{W}_i + \frac{\lambda_c}{\tau\sigma_h^2}\boldsymbol{m}_{c,t} \cdot \Big(\big(\boldsymbol{W}_p \cdot \tanh(\boldsymbol{m}_{h,t}^{post})\big) \odot \big(\boldsymbol{W}_f \cdot \boldsymbol{\epsilon}_{h,t+1}\big)\Big)^\intercal \tag{60}$$

B. Parameters

**Model**	**Parameters**
Parameters common to all models that are not optimized	$d_h = 300$<br>$d_x = 2$<br>$d_c = p$ (when relevant)<br>$\alpha_x = 10^{-5}$ (when relevant)<br>$\alpha_h = 0$ (when relevant)<br>$\tau = 50$
ESN	$\lambda = 3,4.10^{-6}$
EWC	$\lambda = 3,2.10^{-6}$<br>$\beta = 1,0$
ESN + GR	$\lambda = 3,0.10^{-5}$
Conceptors	$\lambda = 2.10^{-4}$<br>$\alpha = 3,6$
PC-RNN-V	$\lambda = 2,8.10^{-6}$<br>$\lambda_r = 95$
P-TNCN	$\lambda = 2,8.10^{-6}$<br>$\lambda_b = 2,8.10^{-6}$<br>$\lambda_r = 0,01$
PC-RNN-Hebb	$\lambda = 2,8.10^{-6}$<br>$\lambda_b = 2,8.10^{-6}$<br>$\lambda_r = 0,01$
PC-RNN-HC-A	$\lambda = 1,5.10^{-6}$<br>$\lambda_r = 0,01$<br>$\lambda_c = 6,9.10^{5}$
PC-RNN-HC-M	$\lambda = 7,4.10^{-6}$<br>$\lambda_r = 0,01$<br>$\lambda_c = 9,1.10^{4}$
PC-RNN-HC-A-RS	$\lambda = 5,6.10^{-6}$<br>$\lambda_r = 91$<br>$\sigma = 10^{-3}$
PC-RNN-HC-M-RS	$\lambda = 2,8.10^{-6}$<br>$\lambda_r = 10^{-4}$<br>$\sigma = 10^{-8}$
PC-RNN-HC-A + Conceptors	$\lambda = 1,2.10^{-4}$<br>$\lambda_r = 0,1$<br>$\lambda_c = 1,4.10^{5}$<br>$\alpha = 5,0$
PC-RNN-HC-A + GR	$\lambda = 3,0.10^{-5}$<br>$\lambda_r = 90$<br>$\lambda_c = 5,0.10^{5}$

Table 3: Hyperparameters for the continual learning benchmark.
